# Adding Suicide Prevention to the Triple Advantages of Injectable Long-Acting Second-Generation Antipsychotics

**DOI:** 10.3389/fpsyt.2019.00931

**Published:** 2020-01-14

**Authors:** Maurizio Pompili

**Affiliations:** Department of Neuroscience, Mental Health and Sensory Organs, Suicide Prevention Center, Sant'Andrea Hospital, Sapienza University of Rome, Rome, Italy

**Keywords:** suicide, treatmeat outcome, relapse, adherence, prevention

## Introduction

With the advent of new treatment perspectives for schizophrenic patients, long-acting injectable antipsychotics promise to provide relapse prevention, neuroprotection, and lower mortality rates (1). In preliminary studies, scholars have also indicated that such treatments could play a central role in suicide prevention in patients (2, 3) for whom suicide is the most likely cause of premature death, in addition to repercussions for caregivers and clinicians. Both Kraepelin (4) and Bleuler (5) highlighted the issue of suicide risk among patients suffering from schizophrenia. Modern studies provided an intricate list of risk factors for suicide, pointing to numerous issues in the management and treatment of schizophrenic patients. Individuals at higher risk are generally unmarried young white males who achieved proper functioning before the onset of the disorder (6). Such patients may easily be overwhelmed by hopelessness and depression, as well as becoming demoralized and being aware that their previous lifestyle cannot be maintained. Such patients feel socially isolated, fear further mental deterioration, have higher rates of substance abuse, and may lose faith in the treatments. Suicide attempts and hospitalizations are frequent, and each time they may realize that both medical and family support is limited; moreover, *ad hoc* interventions are not available or are difficult to implement. There are also further risk factors for suicide in schizophrenia, such as post-psychotic depression, agitation or motor restlessness, poor adherence to treatment, and command hallucinations (although not well ascertained in terms of statistical significance). However, risk factors often yield too many false positives, pointing to the need for suicide assessments based on factors such as an understanding of mental pain and demoralization, as well as therapeutic relationships, foreseeable life events that may impact the patient, social support, and the available resources. Furthermore, suicide risk is indirectly related to relapse, illness progression, the number of hospitalizations, and plans for rehabilitation after discharge (7).

Each admission to a psychiatric ward may represent a further loss of hope and faith in the treatments. The risk of suicide seems to peak not only shortly after discharge, as reported routinely in the literature, but also shortly after admission (7). This fact points to the implementation of human–environmental factors for providing ward safety and close supervision in both inpatient and community settings, especially in the cases of reduced adherence to treatment.

Among the various risk factors, preventative actions should be directed over those factors that are modifiable and can be targeted by proper treatment actions (8). Furthermore, suicide risk seems to be higher when patients develop demoralization syndrome, in which repeated exacerbations of psychotic symptoms, functional deterioration compared with premorbid abilities, and a non-delusional awareness of the effects of an illness can lead to feelings of hopelessness, depression, and, ultimately, suicide.

Suicidal behavior is also a major issue among patients with first-episode psychosis (9, 10). It is noteworthy that integrated compared with typical treatment proved to be effective in reducing both suicide and other causes of death; the Opus Study (9) demonstrated the effectiveness of this strategy. Such an intervention involved a) assertive community treatment (ACT); b) antipsychotic medication; c) psychoeducational family treatment; and d) social skills training. It resulted in lower mortality for any cause and lower suicide rates.

### Mortality in Schizophrenia and Pharmacological Treatment

A recent study by Bitter et al. (11) highlighted the reduction in life expectancy among patients with schizophrenia. Of note is the fact that, compared with the controls, 20-year-old males with schizophrenia had their life expectancy reduced by 11.5 years and females by 13.7 years; the analogous numbers for 45-year-old schizophrenics were 8.1 and 9.6 years, respectively.

Taipale et al. (12) explored mortality in a large sample of approximately 30,000 patients and found that second-generation long-acting antipsychotic therapies [SG LATs; ranking: paliperidone palmitate > oral aripiprazole > long-acting injection (LAI) risperidone > LAI haloperidol > LAI perphenazine > oral perphenazine > LAI olanzapine > LAI zuclopenthixol] were associated with the lowest cumulative mortality rate. In 7.5 years, a follow-up SG LAT observation revealed the lowest cumulative mortality rate as compared with first-generation (FG) and SG oral and with no antipsychotic use. The use of LATs reduces the risk of death by 33% when compared to the corresponding oral equivalent. Such results are in line with further investigations by the same group of researchers (13), who reported their observations over 8 years. They observed that the risk of treatment failure or relapse after discontinuing antipsychotics does not decrease over time and that patients maintaining long-term antipsychotic treatment had increased survival rates.

### Pharmacological Treatment and Outcome

Most of the challenges in managing schizophrenia are connected to relapses (a variable correlated with increased time to remission, increased residual symptom severity, increased risk of treatment resistance, increased cerebral toxicity, cognitive decline and functional impairment, increased risk of mortality, and increased duration of time to remission).

In this regard, suicide risk has been associated with reduced adherence to treatment and relapse rates (14). Seminal papers (15, 16) indicated that extended periods of relapse in schizophrenia might harm brain integrity. Evidence supports the notion that the risk increases immediately after stopping antipsychotics and remains high over time; on the other hand, maintaining antipsychotic treatment is associated with a reduced risk of relapse, fewer hospitalizations, and a better quality of life compared with a placebo or no treatment.

Heres et al. (17) pointed out the risks of using antipsychotic medications inappropriately as related to an increased risk of symptom worsening or relapse. Zipursky et al. (18), investigating the 1-year mean risk of psychotic symptoms in patients who have remitted after their first episode, found that recurrence after medication discontinuation is 77% compared to 3% under maintenance treatment. Each time, the exacerbation of symptoms is associated with mental pain and hopelessness. The concept of mental pain is of great importance as suicide may be considered an escape from intolerable suffering, emphasizing that suicide is not a movement toward death but rather an escape from pain and unendurable or unacceptable anguish. Experiencing negative emotions alongside an internal dialogue causes a painful flow of consciousness and leads the individual to the ultimate conclusion: death. This may be related to the fact that, if tormented individuals could somehow stop their consciousness and remain alive, they would opt for that solution. We may, therefore, hypothesize that proper pharmacological treatment targets those conditions which otherwise cause the individual to suffer, feel hopeless, and go on to die by suicide. It is, therefore, not necessarily the emergence of specific symptoms in each relapse, but the impact of the vivid reflection of the course of illness that potentiates suicide risk.

Of interest are the results reported by Hui et al. (19). These authors found that over 10 years, poor clinical outcomes occurred in 39% of patients who had interrupted their treatments versus 21% of patients in the maintenance treatment group. Incidentally, suicide was the only serious adverse event that occurred in the follow-up phase. This study also reveals that first-episode psychosis with a full initial response to treatment and medication continuation for at least the first 3 years after starting treatment decreases the risk of relapse and poor long-term clinical outcomes.

The number of hospitalizations is a central risk factor for suicide as each admission is indicative of poor outcomes, a painful awareness of ineffective treatment, and a fear of further mental disintegration. Kishimoto et al. (20) demonstrated that treatment with LAIs is associated with a reduced risk of hospitalization. Considering that such a reduction may increase patients' faith in treatment, as well as their faith in the future, this treatment may protect patients against suicide risk. In a substantial sample comprising almost 30,000 patients, Tiihonen et al. (21) found that treatments with some of the LAIs available (ranking: LAI paliperidone > LAI zuclopenthixol > oral clozapine > LAI perphenazine > LAI olanzapine > LAI risperidone) led to a reduction in hospitalizations. Furthermore, clozapine and LAIs were found to be the best treatment for relapse prevention.

In a pilot study, recently diagnosed patients with schizophrenia showed more significant improvements versus patients diagnosed for more than 5 years in an adjusted mean Global Assessment of Functioning (GAF) score, Positive and Negative Syndrome Scale (PANSS) factor score for negative and depressive symptoms, and the severity and intensity of their suicidal ideation (patients were treated with LAI-SGA during a follow-up period of 12 months). Our preliminary findings support the hypothesis that long-acting injection second-generation antipsychotics (LAI-SGA) may influence the course of the illness if administered in the early phase of the illness (3).

## Discussion

Recently, Stahl (22) challenged out the traditional association between LAI formulations of antipsychotics and patients with schizophrenia with the most severe symptoms, in addition to those with the poorest adherence to treatment, numerous hospitalizations, and chronicity of the disorder. On the other hand, LAI antipsychotics may represent a strategic treatment in early-episode patients, resulting in the administration of treatments when schizophrenia is most treatable. In turn, such an approach may reduce recurrences and rehospitalizations. Since the first hospitalization is usually a crucial turning point for influencing the outcome, proper innovative treatment with LAI antipsychotics may make the difference in the moderation of a number of key risk factors for suicide in schizophrenia.

Aside from proper pharmacological properties, LAIs also possess several other advantages over oral antipsychotics, including those that may provide suicide prevention; they are administered by a mental health professional, and so, therefore, have a secondary gain which is shared by lithium and clozapine. In fact, in such treatments (both with consolidating evidence of their anti-suicidal action), patients need to regularly check their lithium blood levels; the same applies to clozapine, for which patients need to have regular blood count check to avoid agranulocytosis. Such small actions have the potential to strengthen therapeutic relationships and, in turn, diminish the suicide risk, as well as increase therapeutic contact and verify patients' adherence to treatment.

In summary, apart from being associated with the lowest cumulative mortality rate, SG LATs have the potential to influence various risk factors for suicide among patients with schizophrenia. The improvement of patients' functioning is of great importance as patients may consider suicide, especially during phases with the absence of positive and negative symptoms. The experience of painful insights, social isolation, and a fear of further mental deterioration associated with any condition contributing to patients' mental pain seem to be related to the risk of suicide. Appropriate treatment, as in the case of LAIs, which ensures the same concentration over time, may mitigate aggression and impulsivity, as well as substance abuse.

It is essential to take advantage of the benefits LAIs offer by combining drug treatment with psychosocial–psychotherapeutic interventions for a better outcome. Such patients can benefit from programs involving a psychotherapist as a critical figure in order to overcome painful periods, as well as be able to better cope with their conflicts. Clinicians should acknowledge the patient's despair, discuss their losses and daily difficulties, and help them to establish new and accessible goals.

Future studies should comprehensively assess how much injectable long-acting antipsychotics can affect suicide rates among patients with schizophrenia. Nowadays, it seems clinically reasonable to conclude that such treatments are promising in their management of symptoms more associated with suicide risk.

## Author Contributions

The author confirms being the sole contributor of this work and has approved it for publication.

## Conflict of Interest

The author wishes to disclose that in the last two years he has lectured and served on advisory board honoraria or engaged in clinical trial activities with Angelini, Lundbeck, Janssen, Otsuka, and Allergan.

## References

[1] Nasrallah HA Triple advantages of injectable long acting second generation antipsychotics: relapse prevention, neuroprotection, and lower mortality. Schizophr Res (2018) 197:69–70. 10.1016/j.schres.2018.02.004 29506767

[2] Pompili M Orsolini L Lamis DA Goldsmith DR Nardella A Falcone G Suicide prevention in schizophrenia: do Long-Acting Injectable antipsychotics (LAIs) have a role? CNS Neurol Disord Drug Targets (2017) 16:454–62. 10.2174/1871527316666170223163629 28240189

[3] Corigliano V Comparelli A Mancinelli I Montalbani B Lamis DA De Carolis A Long-acting injectable second-generation antipsychotics improve negative symptoms and suicidal ideation in recent diagnosed schizophrenia patients: a 1-year follow-up pilot study. Schizophr Res Treat (2018) 30. 10.1155/2018/4834135 PMC6136552 30245878

[4] Kraepelin E Dementia praecox and paraphrenia. Livingstone: Edimburgh (1919).

[5] Bleuler E Dementia praecox or the group schizophrenias. New York: International University Press (1950).

[6] Pompili M Amador XF Girardi P Harkavy-Friedman J Harrow M Kaplan K Suicide risk in schizophrenia: learning from the past to change the future. Ann Gen Psychiatry (2007) 6:10. 10.1186/1744-859X-6-10 17367524 PMC1845151

[7] Pompili M Mancinelli I Ruberto A Kotzalidis GD Girardi P Tatarelli R Where schizophrenic patients commit suicide a review of suicide among inpatients and former inpatients. Int J Psychiatry Med (2005) 35:171–90. 10.2190/9CA1-EL73-1VXD-9F2V 16240974

[8] Pompili M Lester D Innamorati M Tatarelli R Girardi P Assessment and treatment of suicide risk in schizophrenia. Expert Rev Neurother (2008) 8:51–74. 10.1586/14737175.8.1.51 18088201

[9] Nordentoft M Jeppesen P Abel M Kassow P Petersen L Thorup A OPUS study: suicidal behaviour, suicidal ideation and hopelessness among patients with first-episode psychosis. One-year follow-up of a randomised controlled trial. Br J Psychiatry (2002) Suppl 43:98–106. 10.1192/bjp.181.43.s98 12271808

[10] Pompili M Serafini G Innamorati M Lester D Shrivastava A Girardi P Suicide risk in first episode psychosis: a selective review of the current literature. Schizophr Res (2011) 129:1–11. 10.1016/j.schres.2011.03.008 21530179

[11] Bitter I Czobor P Borsi A Fehér L Nagy BZ Bacskai M Mortality and the relationship of somatic comorbidities to mortality in schizophrenia. A nationwide matched-cohort study. Eur Psychiatry (2017) 45:97–103. 10.1016/j.eurpsy.2017.05.022 28753464

[12] Taipale H Mittendorfer-Rutz E Alexanderson K Majak M Metala J Hoti F Antipsychotics and mortality in a nationwide cohort of 29,823 patients with schizophrenia. Schizophr Res (2018) 197:274–80. 10.1016/j.schres.2017.12.010 29274734

[13] Tiihonen J Tanskanen A Taipale H 20-Year nationwide follow-up study on discontinuation of antipsychotic treatment in first-episode schizophrenia. Am J Psychiatry (2018) 175(8):765–73. 10.1176/appi.ajp.2018.17091001 29621900

[14] Pompili M Serafini G Del Casale A Rigucci S Innamorati M Girardi P Improving adherence in mood disorders: the struggle against relapse, recurrence and suicide risk. Expert Rev Neurother (2009) 9:985–1004. 10.1586/ern.09.62 19589049

[15] Andreasen NC Liu D Ziebell S Vora A Ho BC Relapse duration, treatment intensity, and brain tissue loss in schizophrenia: a prospective longitudinal MRI study. Am J Psychiatry (2013) 170:609–15. 10.1176/appi.ajp.2013.12050674 PMC3835590 23558429

[16] Emsley R Chiliza B Asmal L Harvey BH The nature of relapse in schizophrenia. BMC Psychiatry (2013) 8:13–50. 10.1186/1471-244X-13-50 PMC3599855 23394123

[17] Heres S Lambert M Vauth R Treatment of early episode in patients with schizophrenia: the role of long acting antipsychotics. Eur Psychiatry (2014) 29 Suppl 2:1409–13. 10.1016/S0924-9338(14)70001-X 25455704

[18] Zipursky RB Menezes NM Streiner DL Risk of symptom recurrence with medication discontinuation in first-episode psychosis: a systematic review. Schizophr Res (2014) 152:408–14. 10.1016/j.schres.2013.08.001 23972821

[19] Hui CLM Honer WG Lee EHM Chang WC Chan SKW Chen ESM Long-term effects of discontinuation from antipsychotic maintenance following first-episode schizophrenia and related disorders: a 10 year follow-up of a randomised, double-blind trial. Lancet Psychiatry (2018) 5:432–42. 10.1016/S2215-0366(18)30090-7 29551618

[20] Kishimoto T Hagi K Nitta M Leucht S Olfson M Kane JM Effectiveness of long-acting injectable vs oral antipsychotics in patients with schizophrenia: a meta-analysis of prospective and retrospective cohort studies. Schizophr Bull (2018) 44(3):603–19. 10.1093/schbul/sbx090 PMC5890463 29868849

[21] Tiihonen J Mittendorfer-Rutz E Majak M Taipale H Real-world effectiveness of antipsychotic treatments in a nationwide cohort of 29 823 patients with schizophrenia. JAMA Psychiatry (2017) 74:686–3. 10.1001/jamapsychiatry.2017.1322 PMC5710250 28593216

[22] Stahl SM Long-acting injectable antipsychotics: shall the last be first? CNS Spectr (2014) 19:3–5. 10.1017/S1092852913001016 24512639

