# Corrigendum: Adding Suicide Prevention to the Triple Advantages of Injectable Long-Acting Second-Generation Antipsychotics

**DOI:** 10.3389/fpsyt.2020.00101

**Published:** 2020-02-27

**Authors:** Maurizio Pompili

**Affiliations:** Department of Neuroscience, Mental Health and Sensory Organs, Suicide Prevention Center, Sant'Andrea Hospital, Sapienza University of Rome, Rome, Italy

**Keywords:** suicide, treatment outcome, relapse, adherence, prevention

The author wishes to disclose that in the last two years he has lectured and served on advisory board honoraria or engaged in clinical trial activities with Angelini, Lundbeck, Janssen, Otsuka, and Allergan. 

The author apologizes for this error and states that this does not change the scientific conclusions of the article in any way. The original article has been updated.

